# The Associations of Suspected COVID-19 Symptoms with Anxiety and Depression as Modified by Hemodialysis Dietary Knowledge: A Multi-Dialysis Center Study

**DOI:** 10.3390/nu14122364

**Published:** 2022-06-07

**Authors:** Loan T. Dang, Thuc C. Luong, Dung H. Nguyen, Trung A. Hoang, Hoai T. Nguyen, Hoang C. Nguyen, Thai H. Duong, Tu T. Tran, Linh V. Pham, Tuan V. Ngo, Hoi T. Nguyen, Nga T. Trieu, Thinh V. Do, Manh V. Trinh, Tung H. Ha, Dung T. Phan, Binh N. Do, Shwu-Huey Yang, Tsae-Jyy Wang, Tuyen Van Duong

**Affiliations:** 1 School of Nursing, National Taipei University of Nursing and Health Sciences, Taipei 112-19, Taiwan; dangthiloan@hmu.edu.vn; 2 Faculty of Nursing and Midwifery, Hanoi Medical University, Hanoi 115-20, Vietnam; 3 Director Office, Military Hospital 103, Hanoi 121-08, Vietnam; luongcongthuc@vmmu.edu.vn; 4 Department of Cardiology, Cardiovascular Center, Military Hospital 103, Hanoi 121-08, Vietnam; 5 Hemodialysis Department, Nephro-Urology-Dialysis Center, Bach Mai Hospital, Hanoi 115-19, Vietnam; nhdungbm@gmail.com (D.H.N.); hoangtrung.doctor@gmail.com (T.A.H.); 6 International Ph.D. Program in Medicine, College of Medicine, Taipei Medical University, Taipei 110-31, Taiwan; d142109017@tmu.edu.tw; 7 Division of Military Scientific Information, Vietnam Military Medical University, Hanoi 121-08, Vietnam; hoaihvqy@gmail.com; 8 Director Office, Thai Nguyen National Hospital, Thai Nguyen 241-24, Vietnam; nguyenconghoang@tnmc.edu.vn (H.C.N.); dhthaivn@gmail.com (T.H.D.); 9 President Office, Thai Nguyen University of Medicine and Pharmacy, Thai Nguyen 241-17, Vietnam; 10 Department of Internal Medicine, Thai Nguyen University of Medicine and Pharmacy, Thai Nguyen 241-17, Vietnam; 11 Department of Pulmonary & Cardiovascular Diseases, Hai Phong University of Medicine and Pharmacy Hospital, Hai Phong 042-12, Vietnam; pvlinh@hpmu.edu.vn; 12 Department of Hemodialysis, Hai Phong University of Medicine and Pharmacy Hospital, Hai Phong 042-12, Vietnam; ngovantuanbs@gmail.com; 13 Director Office, Hai Phong International Hospital, Hai Phong 047-08, Vietnam; hoinguyenthanhbm@gmail.com; 14 Hemodialysis Division, Hai Phong International Hospital, Hai Phong 047-08, Vietnam; bstrieunga@gmail.com; 15 Director Office, Bai Chay Hospital, Ha Long 011-21, Vietnam; dovanthinhhscc@gmail.com; 16 Director Office, Quang Ninh General Hospital, Ha Long 011-08, Vietnam; trinhmanhqnsyt@gmail.com; 17 Director Office, General Hospital of Agricultural, Hanoi 125-16, Vietnam; hahuutung.200564@gmail.com; 18 Faculty of Nursing, Hanoi University of Business and Technology, Hanoi 116-22, Vietnam; phanthidzungvd@gmail.com; 19 Nursing Office, Thien An Obstetrics and Gynecology Hospital, Hanoi 112-06, Vietnam; 20 Department of Infectious Diseases, Vietnam Military Medical University, Hanoi 121-08, Vietnam; nhubinh.do@vmmu.edu.vn; 21 Division of Military Science, Military Hospital 103, Hanoi 121-08, Vietnam; 22 School of Nutrition and Health Sciences, Taipei Medical University, Taipei 110-31, Taiwan; sherry@tmu.edu.tw; 23 Nutrition Research Center, Taipei Medical University Hospital, Taipei 110-31, Taiwan; 24 Research Center of Geriatric Nutrition, Taipei Medical University, Taipei 110-31, Taiwan

**Keywords:** hemodialysis, psychometric properties, anxiety, depression, suspected COVID-19 symptoms, health literacy, digital healthy diet literacy, hemodialysis dietary knowledge, Vietnam

## Abstract

During the COVID-19 pandemic, it is essential to evaluate hemodialysis patients’ dietary knowledge, especially among those with COVID-19 related symptoms, in order to identify appropriate strategies in managing their mental health. The study’s purposes were to test the psychometric properties of the hemodialysis dietary knowledge (HDK) scale, and to investigate the modifying impact of HDK on the associations of suspected COVID-19 symptoms (S-COVID-19-S) with anxiety and depression among hemodialysis patients. A cross-sectional study was conducted from July 2020 to March 2021 at eight hospitals across Vietnam. Data of 875 hemodialysis patients were analyzed, including socio-demographic, anxiety (the generalized anxiety disorder scale, GAD-7), depression (the patient health questionnaire, PHQ-9), S-COVID-19-S, HDK, health literacy, and digital healthy diet literacy. Confirmatory factor analysis (CFA) and logistic regression models were used to analyze the data. The HDK scale demonstrates the satisfactory construct validity with good model fit (Goodness of Fit Index, GFI = 0.96; Adjusted Goodness of Fit Index, AGFI = 0.90; Standardized Root Mean Square Residual, SRMR = 0.05; Root Mean Square Error of Approximation, RMSEA = 0.09; Normed Fit Index, NFI = 0.96; Comparative Fit Index, CFI = 0.96, and Parsimony goodness of Fit Index, PGFI = 0.43), criterion validity (as correlated with HL (r = 0.22, *p* < 0.01) and DDL (r = 0.19, *p* < 0.01), and reliability (Cronbach alpha = 0.70)). In the multivariate analysis, S-COVID-19-S was associated with a higher likelihood of anxiety (odds ratio, OR, 20.76; 95% confidence interval, 95%CI, 8.85, 48.70; *p* < 0.001) and depression (OR, 12.95; 95%CI, 6.67, 25.14, *p* < 0.001). A higher HDK score was associated with a lower likelihood of anxiety (OR, 0.70; 95%CI, 0.64, 0.77; *p* < 0.001) and depression (OR, 0.72; 95%CI, 0.66, 0.79; *p* < 0.001). In the interaction analysis, the negative impacts of S-COVID-19-S on anxiety and depression were mitigated by higher HDK scores (*p* < 0.001). In conclusion, HDK is a valid and reliable tool to measure dietary knowledge in hemodialysis patients. Higher HDK scores potentially protect patients with S-COVID-19-S from anxiety and depression during the pandemic.

## 1. Introduction

Anxiety and depression are highly prevalent in patients with end-stage renal disease (ESRD) undergoing hemodialysis [1]. The prevalence varies (from 22.5% to nearly 85% for depression; from 32.3% to 44.7% for anxiety) by populations and measurement tools [2,3,4,5,6]. In hemodialysis patients, anxiety and depression have been linked to adverse outcomes such as lower quality of life, poorer clinical parameters (e.g., loss of vascular access, poorer nutritional status) [3,4], reduced treatment adherence [7], increased risk for death or hospitalization [8,9,10], and more common suicidal ideation [11,12]. However, anxiety and depression remain under-recognized and neglected in ESRD patients [13,14].

Amidst the COVID-19 pandemic, hemodialysis patients are highly vulnerable to COVID-19 infection [14]. Prevalence of COVID-19 infection in hemodialysis patients ranged from a lower rate, such as 5.3% in Qatar [15], to higher rates, such as 19% in Paris [16] and 36.2% in the United Kingdom [17]. The incidence of COVID-19 and mortality rate in these patients was 7.7% and 22.4%, respectively [18]. Multiple studies have examined the factors associated with depression and anxiety among hemodialysis patients prior to the COVID-19 pandemic [19,20,21,22] and during such context [23,24]. However, there has been a lack of studies investigating the association between COVID-19 related symptoms and mental health in hemodialysis patients. Meanwhile, previous studies found that having S-COVID-19-S was associated with higher odds of anxiety and depression in another population [25,26].

Insufficient dietary or nutritional knowledge was found in patients receiving hemodialysis [27]. In addition, people tended to change their eating habits and dietary intake during the COVID-19 pandemic [28,29]. Having adequate hemodialysis dietary knowledge (HDK) may help ESRD patients in selecting healthy foods and maintaining a healthy dietary intake, which was found to modify the negative effect of COVID-19 lockdown on depression in outpatients [30].

It is important to have a valid and reliable tool to assess dietary knowledge in hemodialysis patients, and explore its role in mental health, especially in those with suspected COVID-19 symptoms (S-COVID-19-S). Therefore, we aimed to test the psychometric properties of the hemodialysis dietary knowledge (HDK) scale, and to investigate the modifying impact of HDK on the associations of S-COVID-19-S with anxiety and depression among hemodialysis patients. We hypothesized that hemodialysis patients with S-COVID-19-S have a higher likelihood of anxiety and depression than those without symptoms, whereas patients with a higher HDK score have a lower likelihood of anxiety and depression, especially in those with S-COVID-19-S. 

## 2. Materials and Methods

### 2.1. Study Design and Sample

A cross-sectional study was conducted from July 2020 to March 2021. Participants were recruited from dialysis centers in eight hospitals in Vietnam. The study was reviewed and approved by the Institutional Ethical Review Committee of Hanoi University of Public Health, Vietnam (IRB No. 225/2020/YTCC-HD3).

A total sample of 1048 participants was invited to participate in the study, including from Bach Mai Hospital (*n* = 251), Military Hospital 103 (*n* = 147), General Hospital of Agriculture (*n* = 171), Thai Nguyen National Hospital (*n* = 170), Hai Phong University of Medicine and Pharmacy Hospital (*n* = 82), Hai Phong International Hospital (*n* = 43), Quang Ninh General Hospital (*n* = 103), and Bai Chay Hospital (*n* = 81). Patients recruited were those aged 18–85 years, receiving hemodialysis treatment for at least three months, and able to read and speak Vietnamese. Patients excluded from final analysis were those with hemodialysis vintage less than 3 months (*n* = 56), age <18 years or >85 years (*n* = 20), pregnant (*n* = 1), amputation (*n* = 1), tube feeding (*n* = 1), having a scheduled surgery (*n* = 2), and having arteriovenous fistula (AVF) dysfunction (*n* = 92). The final sample of 875 patients was analyzed. This sample was large enough for the analysis in our study as it is larger than the required sample of 261, calculated using G*Power software Version 3.1.9.7 (Heinrich-Heine-Universität, Düsseldorf, Germany) [31] with an effect size of 0.05, type I error of 0.05, and power of 0.95, and the proportion of patients with S-COVID-19-S with depression was 0.643 [26]. 

### 2.2. Measurements

#### 2.2.1. Suspected COVID-19 Symptoms

The face-to-face interviews were conducted on patients in the hemodialysis departments. At the time of data collection, participants were screened for S-COVID-19-S and classified as having S-COVID-19-S if they had any of the following symptoms: fever, cough, dyspnea, myalgia, fatigue, sputum production/expectoration, confusion, headache, sore throat, running nose, chest pain, rhinorrhea, diarrhea, or nausea/vomiting [32].

#### 2.2.2. Anxiety

We used the Generalized Anxiety Disorder Scale (GAD-7) to measure anxiety [33]. Participants were asked about and rated how often they have been bothered by each of the seven symptoms during the last two weeks on a 4-point scale (0 = not at all; 1 = several days; 2 = more than half of the days; and 3 = nearly every day). The reliability and validity of this scale have been tested [33,34]. The tool was also validated and used in Vietnam [35,36]. A score of 8 or higher was defined as anxiety [37]. 

#### 2.2.3. Depression

The Patient Health Questionnaire (PHQ-9) was used to measure depression severity [38]. The tool was validated and used in Vietnam [35,39]. It is a valid and reliable tool [40] used in the hemodialysis population [34,41]. Patients were asked to self-report each depressive symptom they had experienced over the last two weeks on a 4-point Likert scale from 0 (not at all) to 3 (nearly every day). The PHQ-9 score ranged from 0 to 27. A score of 10 or higher was classified as depression [38]. 

#### 2.2.4. Hemodialysis Dietary Knowledge

Hemodialysis dietary knowledge (HDK) was used for assessment. The tool was developed and used in a previous study [42]. The HDK scale consists of ten questions related to knowledge of protein, potassium, phosphorus, sodium, and water, each containing two sub-questions [42]. There are three response options, including “correct”, “incorrect”, and “not sure”. The correct answer was treated as “correct”, and incorrect or “not sure” answers were treated as “incorrect”. The possible total score ranges from 0 to 10; a higher score presents better knowledge. The continuous scale was used in a previous study [42] and in this one.

#### 2.2.5. Covariates

In this study, socio-demographics, comorbidity, physical activity, health literacy, and digital health literacy were also included and treated as covariates, which showed associations with mental health in previous studies [26,43]. 

Information related to participants was collected, including age, gender, marital status, education, occupation, social status, ability to pay for medication, body height (cm), weight after dialysis (kg), body mass index (BMI, kg/m^2^), and hemodialysis vintage (years). The Charlson Comorbidity Index (CCI) was used to assess comorbidities [44].

We used the International Physical Activity Questionnaire short version (IPAQ-SF) to assess physical activity (PA) level [45]. Patients were asked to provide information about their time spent in sitting, walking, moderate and vigorous physical activities during the last seven days. The IPAQ was used in the Vietnamese population [46,47]. The overall PA score was calculated by multiplying minutes spent on activities at different levels including sitting, walking, moderate and vigorous activity, by 1.0, 3.3, 4.0, and 8.0, respectively [45,47]. The metabolic equivalent task scored in minutes per week (MET-min/wk) was used as the measuring unit of PA [48].

We evaluated participants’ ability in accessing, understanding, appraising, and applying health-related information and digital healthy-diet-related information using the valid and reliable 12-item short form health literacy questionnaire (HLS-SF12) [49] and digital healthy diet literacy (DDL) [50], respectively. Patients were requested to rate their perceived difficulty of each item using 4-point Likert scales ranging from very difficult (equal to 1) to very easy (equal to 4). The HL and DDL index were standardized to unified metrics from 0 to 50 using the formula mentioned in previous studies [26,50]. Higher HL or DDL index scores indicate a better HL or DDL level [50,51].

### 2.3. Data Collection Procedure

Data collection was conducted by research assistants (doctors, nurses, and medical students). All data collectors were trained in data collection over 4 h by two senior researchers at each hospital. Safety guidance for prevention and control of COVID-19 was also provided during the training, including wearing masks, washing hands, and maintaining physical distance [52]. 

Qualified patients were invited to participate in the survey. Research assistants contacted and asked for voluntary participation. Consent form was achieved before the survey. The face-to-face interviews were conducted at the hemodialysis departments. Adequate time was given to patients. It took about 20–30 min to complete the questionnaire. After the interview, research assistants reviewed the medical records for clinical and biochemical parameters. Finally, all the data were coded, cleaned, and analyzed confidentially by the researchers.

### 2.4. Data Analysis

#### 2.4.1. Psychometric Properties of the HDK

Confirmatory factor analysis (CFA) of the HDK was assessed using Linear Structure Relations (LISREL) Software, version 8.8 (Scientific Software International, Inc., Charlottesville, VA, USA) [53]. Model fit indices were reported, including Goodness of Fit Index (GFI), Adjusted Goodness of Fit Index (AGFI), Standardized Root Mean Square Residual (SRMR), Root Mean Square Error of Approximation (RMSEA), Normed Fit Index (NFI), Comparative Fit Index (CFI), and Parsimony Goodness of Fit Index (PGFI). The index values for adequate model fit were RMSEA < 0.08, GFI > 0.95, AGFI > 0.90, SRMR < 0.05, NFI > 0.90, CFI ≥ 0.95, and PGFI ≤ 0.50 [54,55]. In addition, the correlations among HDK, HL, and DDL were estimated using Pearson correlations to provide evidence of criterion validity [56]. Correlations between the HDK scale and its ten items were estimated using Spearman’s correlation test, which confirmed item-scale convergent validity. The floor and ceiling effects were checked by calculating the percentages of the potential lowest and highest HDK scores. Minimal percentages (<15%) were recommended to eliminate floor and ceiling effects [57]. Internal consistency reliability was assessed using Cronbach’s alpha statistic, with acceptable values of alpha above 0.70 [58]. 

#### 2.4.2. Associated Factors for Anxiety, Depression and Effect Modification of HDK

Descriptive analysis was used to explore the distribution of studied variables. The independent samples *t*-test, Mann-Whitney test, and chi-square test were used appropriately. Next, the associated factors of anxiety and depression were investigated by using bivariate and multivariate logistic regression models. Age, gender, and factors associated with anxiety and depression at *p* < 0.20 in the bivariate model were selected in the multivariate model [59]. The correlations among the independent variables were also checked by Spearman correlation to avoid multi-collinearity (Table S1).

We used PROCESS 3.5 in SPSS to investigate the modified effect of HDK on the relationship between S-COVID-19-S and anxiety/depression. In order to better understand the simple slope analysis, the figure was used to illustrate the results. The figures were drawn by calculating the expected probability of outcomes (anxiety and depression) at three values of HDK (one standard deviation above the mean, +1 SD, the mean, and one standard deviation below the mean, −1 SD) (Tables S3 and S5). The conditional effects were also used to calculate the odds ratios for the impacts of S-COVID-19-S on anxiety or depression at three values of HDK (Tables S2 and S4). IBM SPSS Version 26.0 (IBM Corp, Armonk, NY, USA) was used to analyze the data. Statistical significance was established at *p* < 0.05.

## 3. Results

### 3.1. Participants’ Socio-Demographics

As shown in Table 1, 54.3% were male, aged 18 to 59 years (61.0%). Overall, the prevalence of anxiety and depression were 34.1% and 41.8%, respectively. The prevalence of anxiety and depression were significantly higher in patients with S-COVID-19-S and varied by different groups regarding social status, medication payment ability, edema, physical activity, comorbidity (*p* < 0.05, Table 1). Additionally, compared to patients who have no anxiety and no depression, the mean scores of HL, DDL, HDK of those with anxiety or depression were significantly lower (*p* < 0.05, Table 1).

### 3.2. Psychometric Properties of Hemodialysis Dietary Knowledge

**Figure 1:**
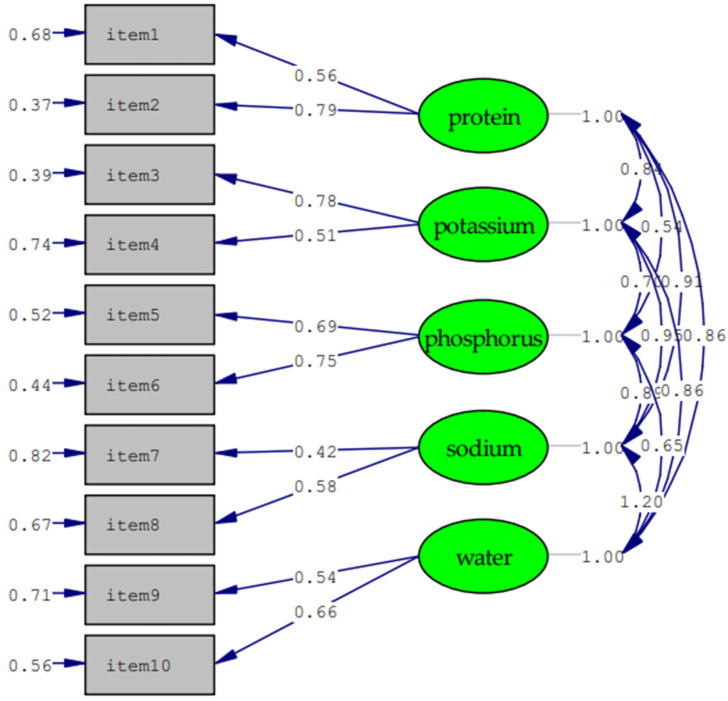
The structure of the hemodialysis dietary knowledge. Parameter estimates are standardized. Model fit indices: GFI = 0.96, AGFI = 0.90, RMSEA = 0.09, SRMR = 0.05, NFI = 0.96, CFI = 0.96, and PGFI = 0.43.

Figure 1 presents the model with standardized estimates. The CFA model resulted in a good fit (except for RMSEA), where GFI = 0.96, AGFI = 0.90, RMSEA = 0.09, SRMR = 0.05, NFI = 0.96, CFI = 0.96, and PGFI = 0.43 (Table 2). The correlations between the HDK scale and its 10 items ranged from 0.37–0.59. In addition, significant positive correlations between HDK and HL, and DDL scores were obtained (r = 0.19 to 0.22, *p* < 0.01). The Cronbach’s alpha value of the HDK for the overall scale was acceptable (α = 0.70). There were no significant floor and ceiling effects (6.17% and 5.26%) (Table 2).

### 3.3. Associated Factors of Anxiety and Depression

In the bivariate analysis, the odds for anxiety and depression were significantly higher in patients with S-COVID-19-S, who were very or fairly difficult to pay for medication, with edema, and with comorbid diseases (*p* < 0.05). By contrast, the odds for anxiety and depression were significantly lower in patients who had more physical activity, with higher HL, DDL, HDK, and with middle or high social status (*p* < 0.05). 

The multivariate analysis illustrated that patients with S-COVID-19-S were more likely to have anxiety (odds ratio, OR, 20.76; 95% confidence interval, 95%CI, 8.85, 48.70, *p* < 0.001), and depression (OR, 12.95, 95%CI, 6.67, 25.14, *p* < 0.001). In comparison with patients in tertile 1 of physical activity, those in tertile 2 (OR, 0.31; 95%CI, 0.18, 0.52, *p* < 0.001), or tertile 3 (OR, 0.50; 95%CI, 0.29, 0.87, *p* = 0.014) had a lower score for anxiety. Similarly, patients in tertile 2 of physical activity (OR, 0.21; 95%CI, 0.12, 0.35, *p* < 0.001), or tertile 3 (OR, 0.26; 95%CI, 0.15, 0.45, *p* < 0.001) had lower odds for anxiety compared with those in tertile 1. The odds for anxiety decreased by 0.4% and 30% for one score increment of HL (OR, 0.96; 95%CI, 0.93, 0.98, *p* = 0.001), and HDK (OR, 0.70; 95%CI, 0.64, 0.77, *p* < 0.001), respectively. The odds of depression decreased by 0.3% and 28% for one score increment of HL (OR, 0.97, 95%CI, 0.95, 0.99, *p* = 0.024) and HDK (OR, 0.72; 95%CI, 0.66, 0.79, *p* < 0.001; Table 3), respectively.

### 3.4. Modification Impacts of Hemodialysis Dietary Knowledge

**Figure 2:**
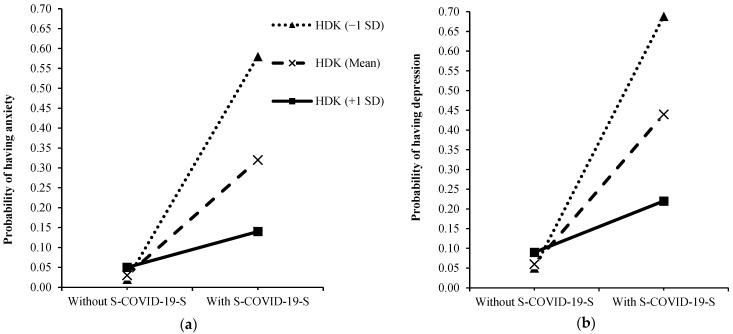
Simple slopes for the interactions between S-COVID-19-S with hemodialysis diet knowledge on anxiety (**a**) and depression (**b**) (*n* = 875). Note: HDK, hemodialysis dietary knowledge; S-COVID-19-S, suspected COVID 19 symptoms; +1 SD, one standard deviation above the mean; −1 SD, one standard deviation below the mean.

Figure 2 illustrates the change in the expected probability of having anxiety and depression with S-COVID-19-S at three levels of HDK (the mean, −1 SD, and +1 SD from the mean). The negative impact of S-COVID-19-S on anxiety was mitigated by higher HDK values from 1 SD below the mean (OR = e^4.4074^ = 82.06; 95%CI, 2.95, 5.87; *p* < 0.001), the mean (OR = e^2.7755^ =16.05; 95%CI, 1.90, 3.65; *p* < 0.001), and 1 SD above the mean (OR = e^1.1436^ = 3.14; 95%CI, 0.06, 2.23; *p* = 0.038). The negative impact of S-COVID-19-S on depression was also mitigated by higher HDK values from 1 SD below the mean (OR = e^3.8368^ = 46.37; 95%CI, 2.83, 4.85; *p* < 0.001), the mean (OR = e^2.4547^ = 11.64; 95%CI, 1.18, 3.10; *p* < 0.001), and 1 SD above the mean (OR = e^1.0726^ = 2.92; 95%CI, 0.19, 1.95; *p* = 0.017).

## 4. Discussion

The simple structure and acceptable psychometric properties of the hemodialysis dietary knowledge (HDK) were established in this study. The construct, criterion validity and reliability of this instrument were acceptable. No floor or ceiling effect of the HDK were confirmed. Through the CFA, goodness-of-fit indices used in this study met the recommended criteria [54]. The CFA supported the model of an original HDK version which consisted of 10 items divided into five components, including protein, potassium, phosphorus, sodium, and water. The overall scale was used in this and a previous study [42]. This study provides the evidence of HDK psychometric properties in Vietnamese context. Further studies are needed to test the validity and reliability of the HDK in different languages and contexts.

In our study, the important predictor of anxiety and depression was S-COVID-19-S. Similarly, previous studies have found that people with S-COVID-19-S had a higher depression likelihood [26,30,60]. In addition, hemodialysis patients who have a family member with suspected or confirmed COVID-19 were more likely to experience anxiety [23]. Hemodialysis patients were more vulnerable to COVID-19 complications because they had been suffering from pre-existing comorbid conditions, which were significantly correlated with mortality [14,61]. Therefore, medical staff should pay more attention to early detection of psychological problems and provide appropriate support to hemodialysis patients, especially to those who have symptoms of COVID-19.

The HDK was found as a protective factor for anxiety and depression. Importantly, our findings also highlighted that HDK alleviated the adverse impact of S-COVID-19-S on hemodialysis patients’ anxiety and depression. This could be explained that having good knowledge about diet may help the hemodialysis patients choose healthy foods, adhering to fluid or dialysis diet prescriptions that may balance the biochemical parameters (e.g., calcium, albumin), and further protect patients’ psychological status [19]. A low nutritional knowledge level in hemodialysis patients has been reported in previous studies [62,63]. In addition, hemodialysis patients who had a higher dietary phosphorus or potassium intake were more likely to increase mortality [64,65], and this was also positively associated with depressed symptoms [66]. Therefore, it is important to have a dietary education program for the improvement of hemodialysis patients’ knowledge and their quality of life [42,67]. Amidst the COVID-19 pandemic, alternative interventions are highly suggested, e.g., dietary mobile applications for hemodialysis patients, and a quick diet and nutrition guide for chronic kidney disease patients [68]. Therefore, we recommended that Vietnamese health care providers, namely dietitians, nephrologists, and nurses, should be aware of providing the necessary knowledge related to healthy diet and nutrition to hemodialysis patients through different methods amidst the pandemic. Further rigorous studies are necessary to examine the effects of enhanced dietary knowledge programs on reducing anxious and depressive symptoms in maintenance hemodialysis patients.

Besides, our finding also demonstrated that hemodialysis patients with S-COVID-19-S and higher HL scores were less likely to be anxious and depressed compared to those people with lower HL scores. This is in line with the findings of previous studies [30,69,70,71]. Increasing HL in patients receiving hemodialysis may improve their quality of life [72], and lower anxiety and depression [73]. However, the literature has documented that hemodialysis patients have limited HL levels [74,75]. Therefore, appropriate strategies should be developed to improve HL in hemodialysis patients. 

In the current study, anxiety and depression were found to be protected by increasing physical activity in hemodialysis patients. This finding is congruent with other studies [30,76]. During the COVID19 pandemic, people tended to have less exercise due to restrictions on outdoor activities, which may further associate with anxiety and depression [77]. Physical activity exerts antidepressant effects through multiple biological and psychosocial pathways [78]. Therefore, regular exercise is highly recommended for hemodialysis patients to prevent psychological consequences during the COVID-19 pandemic. Patients are encouraged to exercise at home or take part in physical activities in the dialysis facilities during hemodialysis sessions. The effects of intradialytic exercise on improving depression and other health outcomes such as quality of life and dialysis adequacy were illustrated in previous studies [79,80]. Lastly, social status was found to be one of the protective factors of anxiety and depression in the present study. Patients with high social status tend to have higher education, higher income, and employment, which were significantly associated with lower levels of depression and anxiety [81].

The present study has some limitations. First, this study was a cross-sectional study that could not determine a direct cause-and-effect association. Future longitudinal or experimental studies are needed to examine the effectiveness of health literacy interventions or diet knowledge education on improving mental health matters in hemodialysis patients. Second, an imbalance probability of S-COVID-19-S resulted in the width of the confidence interval of this variable in relation to anxiety and depression. Third, we only assess knowledge of diet, which may differ from practice. Thus, other tools to measure patients’ eating behaviors are recommended for use in further studies. Finally, some variables such as smoking and drinking that may influence our outcomes have not been studied here.

## 5. Conclusions

The HDK scale was found to be a valid and reliable survey tool for hemodialysis patients in Vietnam. Patients with suspected COVID-19 symptoms had a higher likelihood of having anxiety and depression. Patients with higher HDK scores had a lower likelihood of having anxiety and depression. Importantly, hemodialysis dietary knowledge was found to mitigate the negative impact of S-COVID-19-S on anxious and depressive symptoms. The findings can serve as evidence for developing educational intervention programs, in order to improve patients’ dietary knowledge and health literacy in contributing to the reduction of anxiety and depression.

## Figures and Tables

**Table 1:** Patients’ characteristics, anxiety and depression (*n* = 875).

Variables	Total *n* (%)	GAD < 8 (*n* = 577)	GAD ≥ 8 (*n* = 298)	*p*	PHQ < 10 (*n* = 509)	PHQ ≥ 10 (*n* = 366)	*p*
Age				0.315 ^a^			0.082 ^a^
18–59	534 (61.0)	359 (62.2)	175 (58.7)		323 (63.5)	211 (57.7)	
60–85	345 (39.0)	218 (37.8)	123 (41.3)		186 (36.5)	155 (42.3)	
Gender				0.128 ^a^			0.079 ^a^
Male	475 (54.3)	324 (56.4)	151 (51.0)		289 (57.1)	186 (51.1)	
Female	395 (45.1)	250 (43.6)	145 (49.0)		217 (42.9)	178 (48.9)	
Education				0.840 ^a^			0.905 ^a^
Illiterate or elementary	353 (43.6)	237 (44.1)	116 (42.5)		207 (44.0)	146 (42.9)	
Junior high school	256 (31.6)	170 (31.7)	86 (31.5)		149 (31.7)	107 (31.5)	
Senior high school or above	201 (24.8)	130 (24.2)	71 (26.0)		114 (24.3)	87 (25.6)	
Working status				0.365 ^a^			0.118 ^a^
Not working	311 (35.5)	199 (34.5)	112 (37.6)		170 (33.4)	141 (38.5)	
Working	564 (64.5)	378 (65.5)	186 (62.4)		339 (66.6)	225 (61.5)	
Married status				0.026 ^a^			0.098 ^a^
Never married	76 (8.7)	59 (10.2)	17 (5.7)		51 (10.0)	25 (6.8)	
Ever married	799 (91.3)	518 (89.8)	281 (94.3)		458 (90.0)	341 (93.2)	
Social status				<0.001 ^a^			<0.001 ^a^
Low	233 (26.6)	120 (20.8)	113 (37.9)		99 (19.4)	134 (36.6)	
Middle & high	642 (73.4)	457 (79.2)	185 (62.1)		410 (80.6)	232 (63.4)	
Medication payment ability				<0.001 ^a^			<0.001 ^a^
Very or fairly difficult	660 (76.7)	388 (68.4)	272 (92.5)		328 (65.5)	332 (92.2)	
Very or fairly easy	201 (23.3)	179 (31.6)	22 (7.5)		173 (34.5)	28 (7.8)	
S-COVID-19-S				<0.001 ^a^			<0.001 ^a^
Without S-COVID-19-S	286 (32.7)	248 (43.0)	38 (12.8)		233 (45.8)	53 (14.5)	
With S-COVID-19-S	589 (67.3)	329 (57.0)	260 (87.2)		276 (54.2)	313 (85.5)	
BMI, kg/m^2^				0.478 ^a^			0.049 ^a^
BMI < 24	790 (90.3)	518 (89.8)	272 (91.3)		451 (88.6)	339 (92.6)	
BMI ≥ 24	85 (9.7)	59 (10.2)	26 (8.7)		58 (11.4)	27 (7.4)	
Edema				<0.001 ^a^			<0.001 ^a^
No	467 (53.4)	331 (57.4)	136 (45.6)		305 (59.9)	162 (44.3)	
Yes	408 (46.6)	246 (42.6)	162 (54.4)		204 (40.1)	204 (55.7)	
Hyperthyroidism				0.981 ^a^			0.414 ^a^
No	839 (96.0)	553 (96.0)	286 (96.0)		490 (96.5)	349 (95.4)	
Yes	35 (4.0)	23 (4.0)	12 (4.0)		18 (3.5)	17 (4.6)	
Hospitalization within one month				0.679 ^a^			0.106 ^a^
No	817 (93.5)	537 (93.2)	280 (94.0)		469 (92.3)	348 (95.1)	
Yes	57 (6.5)	39 (6.8)	18 (6.0)		39 (7.7)	18 (4.9)	
Physical activity, MET-min/wk				<0.001 ^a^			<0.001 ^a^
Tertile 1 (MET ≤ 178)	204 (31.6)	105 (23.0)	99 (52.4)		72 (17.6)	132 (55.7)	
Tertile 2 (178 < MET ≤ 960)	224 (34.7)	185 (40.5)	39 (20.6)		175 (42.8)	49 (20.7)	
Tertile 3 (MET > 960)	218 (33.7)	167 (36.5)	51 (27.0)		162 (39.6)	56 (23.6)	
HD vintage, years (Median, IQR)	4.2 (2.1, 7.2)	4.2 (2.0, 7.2)	4.1 (2.6, 7.4)	0.540 ^b^	4.5 (1.9, 7.2)	4.1 (2.6, 7.2)	0.517 ^b^
CCI (Median, IQR)	1.0 (0.0, 2.0)	1.0 (0.0, 2.0)	1.0 (1.0, 4.0)	<0.001 ^b^	1.0 (0.0, 2.0)	1.0 (1.0, 3.0)	<0.001 ^b^
HL index (Mean ± SD)	25.2 ± 9.2	26.8 ± 9.3	22.1 ± 7.9	<0.001 ^c^	26.8 ± 8.9	22.9 ± 9.1	<0.001 ^c^
DDL index (Mean ± SD)	24.1 ± 11.4	25.4 ± 11.7	21.5 ± 10.4	<0.001 ^c^	25.4 ± 11.4	22.3 ± 11.2	<0.001 ^c^
HDK (Mean ± SD)	5.4 ± 2.5	5.9 ± 2.3	4.4 ± 2.67	<0.001 ^c^	5.9 ± 2.3	4.6 ± 2.7	<0.001 ^c^

Abbreviation: S-COVID-19-S, suspected COVID 19 symptoms; BMI, body mass index; MET-min/wk, metabolic equivalent task scored in minutes per week; HD, hemodialysis; CCI, Charlson Comorbidity index; DDL, digital healthy diet literacy; HDK, hemodialysis dietary knowledge. ^a^ Result of chi-square test. ^b^ Result of the Man-Whitney test. ^c^ Result of independent *t* test.

**Table 2:** Psychometric properties of the hemodialysis dietary knowledge Scale (*n* = 875).

HDK Scale	Values
Absolute fit indices	
RMSEA	0.09
GFI	0.96
AGFI	0.90
SRMR	0.05
Incremental fit indices	
NFI	0.96
CFI	0.96
Parsimony fit indices	
PGFI	0.43
Item-scale convergent validity, mean of *rho* ^a^ (range)	0.51 (0.37–0.59)
Criterion validity	
Correlation with HL, *rho* ^b^	0.22 **
Correlation with DDL, *rho* ^b^	0.19 **
Internal consistency, Cronbach’s alpha	0.70
Floor effect, %	6.17
Ceiling effect, %	5.26

Abbreviation: HDK, hemodialysis dietary knowledge; RMSEA, root mean square error of approximation; GFI, goodness of fit index; AGFI, adjusted goodness of fit index; SRMR, standardized root mean square residual; NFI, normed fit index; CFI, comparative fit index; PGFI, parsimony goodness of fit index; HL, health literacy; DDL, digital healthy diet literacy. ^a^ Spearman correlation coefficient. ^b^ Pearson correlation coefficient. **: *p* < 0.01.

**Table 3:** Associated factors of anxiety and depression via bivariate and multivariate logistic regression analysis.

Variables	Anxiety (GAD ≥ 8)				Depression (PHQ ≥ 10)			
	Bivariate		Multivariate		Bivariate		Multivariate	
	OR (95%CI)	*p*	OR (95%CI)	*p*	OR (95%CI)	*p*	OR (95%CI)	*p*
Age								
23–59	1.00		1.00		1.00		1.00	
60–85	1.16 (0.87, 1.54)	0.315	0.85 (0.54, 1.34)	0.484	1.28 (0.97, 1.68)	0.082	1.06 (0.68, 1.65)	0.804
Gender								
Male	1.00		1.00		1.00		1.00	
Female	1.25 (0.94, 1.65)	0.128	0.92 (0.59, 1.44)	0.710	1.28 (0.97, 1.67)	0.079	0.92 (0.60, 1.43)	0.723
Education								
Illiterate or elementary	1.00				1.00			
Junior high school	1.03 (0.74, 1.45)	0.850			1.02 (0.74, 1.41)	0.914		
Senior high school or above	1.12 (0.78, 1.61)	0.556			1.08 (0.76, 1.54)	0.659		
Working status								
Not working	1.00				1.00		1.00	
Working	0.87 (0.65, 1.17)	0.365			0.80 (0.61, 1.06)	0.118	1.32 (0.80, 2.19)	0.275
Married status								
Never	1.00		1.00		1.00		1.00	
Ever	1.88 (1.08, 3.29)	0.026	1.67 (0.65, 4.29)	0.284	1.52 (0.92, 2.50)	0.100	0.99 (0.41, 2.37)	0.980
Social status								
Low	1.00		1.00		1.00		1.00	
Middle or high	0.43 (0.32, 0.59)	<0.001	0.54 (0.33, 0.88)	0.014	0.42 (0.31, 0.57)	<0.001	0.45 (0.27, 0.74)	0.002
Medication payment ability								
Very or fairly easy	1.00				1.00			
Very or fairly difficulty	5.70 (3.57, 9.12)	<0.001			6.25 (4.08, 9.59)	<0.001		
S-COVID-19-S								
Without S-COVID-19-S	1.00		1.00		1.00		1.00	
With S-COVID-19-S	5.16 (3.53, 7.53)	<0.001	20.76 (8.85, 48.70)	<0.001	4.99 (3.56, 7.00)	<0.001	12.95 (6.67, 25.14)	<0.001
BMI, kg/m^2^								
BMI < 24	1.00				1.00		1.00	
BMI ≥ 24	0.84 (0.52, 1.36)	0.478			0.62 (0.38, 0.99)	0.049	0.50 (0.25, 1.00)	0.050
Edema								
No	1.00				1.00			
Yes	1.60 (1.21, 2.12)	<0.001			1.88 (1.43, 2.47)	<0.001		
Hyperthyroidism								
No	1.00				1.00			
Yes	1.01 (0.49, 2.06)	0.981			1.33 (0.67, 2.61)	0.414		
Hospitalization within one month								
No	1.00				1.00		1.00	
Yes	0.89 (0.50, 1.58)	0.679			0.62 (0.35, 1.11)	0.106	0.38 (0.11, 1.36)	0.137
Physical activity, MET-min/wk.								
Tertile 1 (MET ≤ 178)	1.00		1.00		1.00		1.00	
Tertile 2 (178 < MET ≤ 960)	0.22 (0.14, 0.35)	<0.001	0.31 (0.18; 0.52)	<0.001	0.15 (0.10, 0.23)	<0.001	0.21 (0.12; 0.35)	<0.001
Tertile 3 (MET > 960)	0.32 (0.21, 0.49)	<0.001	0.50 (0.29; 0.87)	0.014	0.19 (0.12, 0.29)	<0.001	0.26 (0.15; 0.45)	<0.001
HD vintage	1.01 (0.98, 1.04)	0.670			1.01 (0.97, 1.04)	0.743		
CCI	1.12 (1.07, 1.17)	<0.001			1.10 (1.05, 1.15)	<0.001		
HL index	0.94 (0.93, 0.96)	<0.001	0.96 (0.93, 0.98)	0.001	0.95 (0.94, 0.97)	<0.001	0.97 (0.95, 0.99)	0.024
DDL index	0.97 (0.96, 0.99)	<0.001			0.98 (0.97, 0.99)	<0.001		
HDK	0.79 (0.74, 0.84)	<0.001	0.70 (0.64, 0.77)	<0.001	0.80 (0.76, 0.85)	<0.001	0.72 (0.66, 0.79)	<0.001

Abbreviation: OR, odds ratio; CI, confidence interval; S-COVID-19-S, suspected COVID-19 symptoms; BMI, body mass index; MET-min/wk, metabolic equivalent task scored in minutes per week; HD, hemodialysis; CCI, Charlson Comorbidity index; HL, health literacy; DDL, digital healthy diet literacy; HDK, hemodialysis dietary knowledge.

## Data Availability

Data will be available from the corresponding author on reasonable request.

## Supplementary Materials

The following supporting information can be downloaded at: https://www.mdpi.com/article/10.3390/nu14122364/s1, Table S1: The correlations between independent variables; Table S2: Conditional effect of S-COVID-19-S on anxiety at values of HDK; Table S3: Data for visualizing the conditional effect of S-COVID-19-S on anxiety; Table S4: Conditional effects of S-COVID-19-S on depression at values of HDK; Table S5: Data for visualizing the conditional effect of S-COVID-19-S on depression.
