# Supplementary material for: The Associations of Suspected COVID-19 Symptoms with Anxiety and Depression as Modified by Hemodialysis Dietary Knowledge: A Multi-Dialysis Center Study
Source: Nutrients. 2022 Jun 7;14(12):2364. doi: 10.3390/nu14122364 (PMC9230868; doi:10.3390/nu14122364)
Supplement: Supplementary file 1 [file nutrients-14-02364-s001.zip › nutrients-1702268-supplementary.pdf]

Table S1. The correlations between independent variables

	Age	Gender	Education	Working	Marital	Social	Payment status	S-COVID19-S	BMI	Edema	Hyperthyroidism	Hospitalization	PA	HL	DDL	HDK
Age	1.000															
Gender	.050	1.000														
Education	-.099**	-.065	1.000													
Working	-.063	-.086*	.379**	1.000												
Marital	.205**	.004	-.095**	.153**	1.000											
social	-.022	-.043	.336**	.222**	.035	1.000										
payment	.040	-.047	.182**	.138**	.017	.266**	1.000									
S-COVID19-S	.072*	.086*	.047	.017	.053	-.122**	-.152**	1.000								
BMI	-.009	-.032	.000	-.006	.033	-.003	.066	-.002	1.000							
Edema	.019	.067*	-.113**	.005	.061	-.178**	-.199**	.187**	.018	1.000						
Hyperthyroidism	-.080*	.083*	.030	-.031	-.020	-.048	.012	.055	-.028	-.062	1.000					
Hospitalization	-.012	.001	-.002	-.037	.016	-.093**	-.070*	-.063	.008	.106**	.017	1.000				
PA	-.031	-.096*	.188**	.073	-.006	.242**	**.305****	-.238**	.058	**-.335****	-.006	.029	1.000			
CCI	.162**	.066	-.085*	-.041	.124**	-.150**	-.238**	.267**	.021	**.334****	.035	.098**	**-.406****			
HL	-.144**	-.044	.243**	.193**	-.022	.147**	.230**	-.011	-.030	-.103**	.039	-.024	.185**	1.000		
DDL	-.131**	-.087*	.259**	.164**	-.057	.087*	.178**	-.007	-.027	-.147**	.036	.008	.160**	**.742****	1.000	
HDK	-.024	-.031	-.016	-.025	-.042	.091**	.143**	-.054	-.012	-.176**	.090**	-.014	.081*	.226**	.205**	1.000

Abbreviation: S-COVID-19-S, suspected COVID-19 symptoms; BMI, body mass index; HD, hemodialysis; PA, physical activity; CCI, Charlson Comorbidity index; HL, health literacy; DDL, digital healthy diet literacy; HDK, hemodialysis dietary knowledge.

Table S2. Conditional effect of S-COVID-19-S on anxiety at values of HDK

HDK	Effect	p	LLCI	ULCI
-2.6003 (−1SD)	4.4074	< 0.001	2.95	5.87
.0000 (Mean)	2.7755	< 0.001	1.90	3.65
2.6003 (+1SD)	1.1436	0.0382	0.06	2.23

Abbreviation: S-COVID-19-S, suspected COVID 19 symptoms; HDK, Hemodialysis dietary knowledge; +1SD, one standard deviation above the mean; −1SD, one standard deviation below the mean; LLCI, lower limit confidence interval; ULCI: upper limit confidence interval.

Table S3. Data for visualizing the conditional effect of S-COVID-19-S on anxiety

S-COVID-19-S	HDK	Probability of Anxiety
.0000	-2.6003	.0163
1.000	-2.6003	.5766
.0000	.0000	.0282
1.000	.0000	.3180
.0000	+2.6003	.0484
1.000	+2.6003	.1377

Abbreviation: S-COVID-19-S, suspected COVID 19 symptoms.

Table S4. Conditional effect of S-COVID-19-S on depression at values of HDK

HDK	Effect	p	LLCI	ULCI
-2.6003 (−1SD)	3.8368	< 0.001	2.83	4.85
.0000 (Mean)	2.4547	< 0.001	1.81	3.10
2.6003 (+1SD)	1.0726	0.0167	0.19	1.95

Abbreviation: S-COVID-19-S, suspected COVID 19 symptoms; HDK, Hemodialysis dietary knowledge; +1SD, one standard deviation above the mean; −1SD, one standard deviation below the mean; LLCI, lower limit confidence interval; ULCI: upper limit confidence interval.

Table S5. Data for visualizing the conditional effect of S-COVID-19-S on depression

S-COVID-19-S	HDK	Probability of Anxiety
.0000	-2.6003	.0455
1.000	-2.6003	.6884
.0000	.0000	.0628
1.000	.0000	.4381
.0000	+2.6003	.0806
1.000	+2.6003	.2158

Abbreviation: S-COVID-19-S, suspected COVID 19 symptoms.
